# Corneal nerve loss in patients with TIA and acute ischemic stroke in relation to circulating markers of inflammation and vascular integrity

**DOI:** 10.1038/s41598-022-07353-7

**Published:** 2022-02-28

**Authors:** Adnan Khan, Aijaz Parray, Naveed Akhtar, Abdelali Agouni, Saadat Kamran, Sajitha V. Pananchikkal, Ruth Priyanka, Hoda Gad, Georgios Ponirakis, Ioannis N. Petropoulos, Kuan-Han Chen, Kausar Tayyab, Maher Saqqur, Ashfaq Shuaib, Rayaz A. Malik

**Affiliations:** 1 Department of Medicine, Research Division, Weill Cornell Medicine-Qatar, Doha, Qatar; 2 Department of Neurology and Institute of Neurosciences, Hamad Medical Corporation, Doha, Qatar; 3 Department of Pharmaceutical Sciences, College of Pharmacy, QU Health, Qatar University, Doha, Qatar; 4 Department of Neurology, University of Toronto Mississauga, Mississauga, ON Canada; 5 Stroke Program, Department of Neurology, University of Alberta, Alberta, Canada

**Keywords:** Biomarkers, Neurology

## Abstract

Vascular and inflammatory mechanisms are implicated in the development of cerebrovascular disease and corneal nerve loss occurs in patients with transient ischemic attack (TIA) and acute ischemic stroke (AIS). We have assessed whether serum markers of inflammation and vascular integrity are associated with the severity of corneal nerve loss in patients with TIA and AIS. Corneal confocal microscopy (CCM) was performed to quantify corneal nerve fiber density (CNFD), corneal nerve branch density (CNBD) and corneal nerve fiber length (CNFL) in 105 patients with TIA (n = 24) or AIS (n = 81) and age matched control subjects (n = 56). Circulating levels of IL-6, MMP-2, MMP-9, E-Selectin, P-Selectin and VEGF were quantified in patients within 48 h of presentation with a TIA or AIS. CNFL (P = 0.000, P = 0.000), CNFD (P = 0.122, P = 0.000) and CNBD (P = 0.002, P = 0.000) were reduced in patients with TIA and AIS compared to controls, respectively with no difference between patients with AIS and TIA. The NIHSS Score (P = 0.000), IL-6 (P = 0.011) and E-Selectin (P = 0.032) were higher in patients with AIS compared to TIA with no difference in MMP-2 (P = 0.636), MMP-9 (P = 0.098), P-Selectin (P = 0.395) and VEGF (P = 0.831). CNFL (r = 0.218, P = 0.026) and CNFD (r = 0.230, P = 0.019) correlated with IL-6 and multiple regression analysis showed a positive association of CNFL and CNFD with IL-6 (P = 0.041, P = 0.043). Patients with TIA and AIS have evidence of corneal nerve loss and elevated IL6 and E-selectin levels. Larger longitudinal studies are required to determine the association between inflammatory and vascular markers and corneal nerve fiber loss in patients with cerebrovascular disease.

## Introduction

Cerebrovascular disease is the leading cause of acquired disability and the second leading cause of death in the world^1^. In Qatar, patients with acute stroke are younger than other parts of the world and do not necessarily have the traditional risk factors for stroke^2,3^. There is a need to establish biomarkers that identify people at increased risk of stroke.

Inflammatory cytokines are elevated in acute ischemic stroke^4^. Elevated circulating interleukin-6 (IL-6) is associated with a poorer outcome in patients with acute ischemic stroke (AIS)^5,6^ and predicts recurrent stroke in patients with small vessel disease^7^. Furthermore, elevated levels of matrix metalloproteinase (MMP)-9 are associated with infarct growth and hemorrhagic transformation in patients with acute ischemic stroke^8^, whilst lower levels are associated with a better pre-stroke collateral status^9^. Whilst, vascular endothelial growth factor (VEGF) levels were lower in patients with TIA or AIS^10^, elevated VEGF levels have been associated with larger infarct volume, post stroke cognitive impairment^11^ and poorer outcome^12^. However, a recent systematic review and meta-analysis has shown no association between serum VEGF levels and outcomes in ischemic stroke^13^. Whilst elevated E-selectin levels were independently associated with poorer outcomes in patients with acute ischemic stroke^14^, a recent prospective analysis of the EPIC-Heidelberg study showed no association between E-selectin or P-selectin levels and the risk of incident stroke^15^. These observations suggest a complex temporal relationship between changing levels of circulating markers of inflammation and vascular integrity with outcomes in acute ischemic stroke.

Corneal confocal microscopy (CCM) is a non-invasive ophthalmic imaging technique that has identified corneal nerve loss in patients with diabetic neuropathy^16–18^, other peripheral neuropathies^19^, multiple sclerosis^20,21^ and dementia^22^. Recently, we have shown corneal nerve loss in patients with TIA^23^, acute ischemic stroke^24–27^ and recurrent stroke^28^. Increased plasma adhesion molecules including P-selectin and ICAM-1 predict the development of diabetic neuropathy^29^ and elevated IL-6 has been associated with distal neuropathy in subjects with impaired glucose tolerance^30^.

We have assessed the association between corneal nerve loss and circulating vascular and inflammatory markers in patients with TIA and stroke.

## Results

### Clinical, metabolic and corneal confocal microscopy measures

Clinical, metabolic and CCM parameters in the study participants are given in Table 1.

**Table 1:** Clinical, metabolic, corneal nerve and circulatory markers in study participants.

Characteristics	Controls	TIA	Stroke
Number of participants	56	24	81
Age (years)	47.39 ± 18.23	48.75 ± 10.04	50.36 ± 10.30
BMI (kg/m^2^)	28.14 ± 4.86	27.42 ± 3.30	27.53 ± 4.11
NIHSS Score	NA	**0.54 ± 1.02**	3.27 ± 3.40*
Triglycerides (mmol/l)	1.52 ± 1.28	1.75 ± 0.66	1.54 ± 0.84
Total Cholesterol (mmol/l)	4.99 ± 0.84	5.13 ± 1.55	4.80 ± 1.07
LDL (mmol/l)	3.07 ± 0.75	3.01 ± 1.00	3.15 ± 0.97
HDL (mmol/l)***	**1.27 ± 0.34**	**1.14 ± 0.63**^**‡**^	**0.95 ± 0.25**^**‡**^
BP Systolic (mmHg)***	**128.95 ± 15.12**	**158.38 ± 24.72**^**‡**^	**159.44 ± 29.39**^**‡**^
HbA_1c_ (%)***	**5.53 ± 0.42**	**6.43 ± 1.40**^**‡**^	**6.75 ± 2.14**^**‡**^
CNFL (mm/mm^2^)***	**24.07 ± 4.96**	**17.87 ± 6.52**^**‡**^	**17.01 ± 5.59**^**‡**^
CNFD (no./mm^2^)***	**34.89 ± 6.69**	**30.67 ± 10.29**	**29.14 ± 8.79**^**‡**^
CNBD (no./mm^2^)***	**91.86 ± 44.03**	**56.89 ± 32.65**^**‡**^	**50.08 ± 31.93**^**‡**^
**IL-6 (pg/ml)**	**NA**	**3.12 ± 2.25**	5.79 ± 6.63*
MMP-2 (ng/ml)	NA	156.85 ± 48.36	160.57 ± 46.45
MMP-9 (ng/ml)	NA	148.26 ± 78.87	169.46 ± 74.99
**E-Selectin (ng/ml)**	**NA**	**38.26 ± 17.77**	47.47 ± 18.31*
P-Selectin (ng/ml)	NA	49.71 ± 27.33	51.43 ± 2074
VEGF (pg/ml)	NA	43.11 ± 26.09	39.77 ± 21.93

Results are expressed as mean ± SD. Statistically different results between groups using ANOVA: ***P ≤ 0.001. ^‡^Post hoc results significantly different from controls (P < 0.05). *Statistically different results between patients with TIA and stroke (P < 0.05).

Significant values are in [bold].

**Figure 1:**
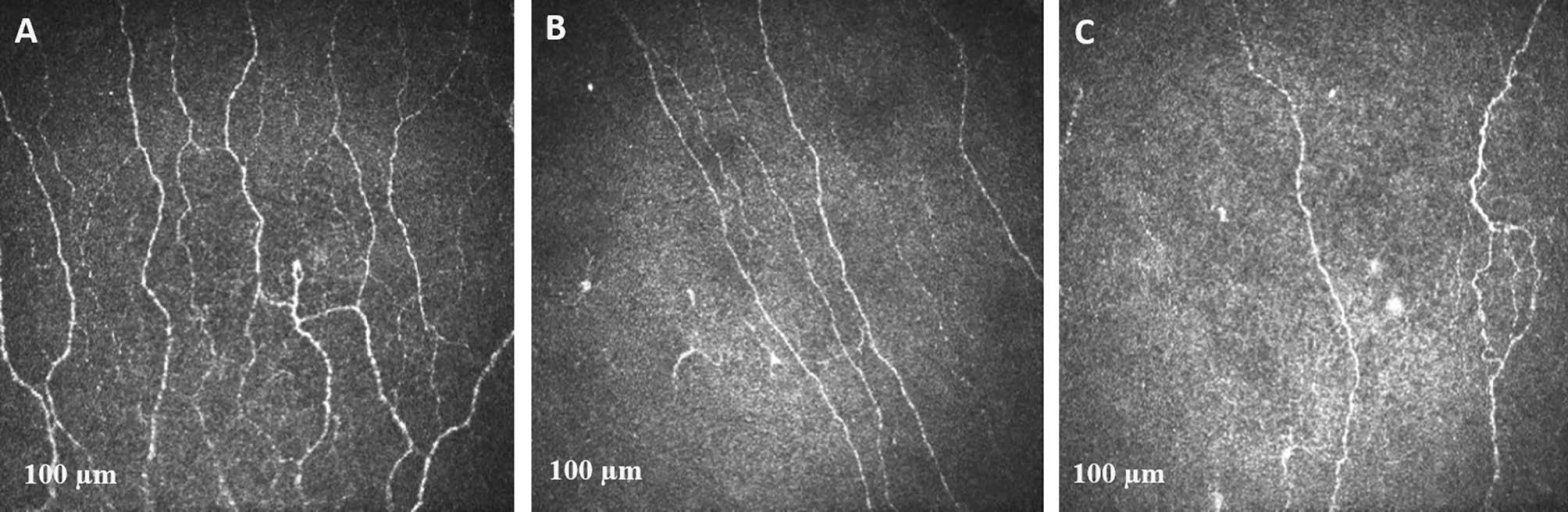
CCM image of the sub-basal nerve plexus in a control participant (**A**) and a patient with TIA (**B**) and an acute ischemic stroke (**C**).

Eighty-one patients with acute ischemic stroke and 24 patients with TIA were compared with 56 age-matched healthy controls. Age (P = 0.634, P = 1.000), BMI (P = 1.000, P = 1.000), triglycerides (P = 1.000, P = 1.000), total cholesterol (P = 1.000, P = 1.000) and LDL (P = 1.000, P = 1.000) in patients with AIS and TIA were comparable to healthy controls. HDL (P = 0.000, P = 0.009), CNFL (P = 0.000, P = 0.000), CNFD (P = 0.000, P = 0.122) and CNBD (P = 0.000, P = 0.002) were lower and systolic BP (P = 0.000, P = 0.000) and HbA_1c_ (P = 0.000, P = 0.002) were higher in patients with AIS and TIA compared to controls with no difference between patients with AIS and TIA (Fig. 1).

The NIHSS Score (P = 0.000), IL-6 (P = 0.011) and E-Selectin (P = 0.032) were higher with no difference in MMP-2 (P = 0.636), MMP-9 (P = 0.098), P-Selectin (P = 0.395) and VEGF (P = 0.831) in patients with AIS compared to TIA.

### Correlation of CCM parameters with inflammatory markers

IL-6 correlated with CNFD (r = 0.230, P = 0.019) and CNFL (r = 0.218, P = 0.026) with no association between corneal nerve parameters and other circulating biomarkers (Table 2).

**Table 2:** Correlation between corneal nerve parameters and circulating markers.

Participants	IL-6 (pg/ml)	MMP-2 (ng/ml)	MMP-9 (ng/ml)	E-selectin (ng/ml)	P-Selectin (ng/ml)	VEGF pg/ml
CNFD, *r*	0.230*	− 0.049	− 0.006	0.031	0.047	0.023
*P*	**0.019**	0.623	0.951	0.751	0.631	0.819
CNFL, *r*	0.218*	− 0.069	0.021	0.059	0.121	0.008
*P*	**0.026**	0.486	0.831	0.551	0.217	0.936
CNBD, *r*	0.075	− 0.012	− 0.049	− 0.003	0.099	0.055
*P*	0.444	0.903	0.622	0.974	0.314	0.574

Significant values are in [bold].

### Multiple linear regression

Multiple regression analysis showed a positive association between CNFL (Table 3) and CNFD (Table 4) with IL-6 and a negative association with age. The CNBD distribution was skewed and was not included in the regression analysis.

**Table 3:** Independent factors for altered CNFL in patients with TIA and stroke.

Dependent variable: CNFL	B	Std. Error	Beta	95% CI Lower bound	95% CI Upper bound	Significance
(Constant)	26.885	5.566		15.824	37.947	0.000
Age (years)	− **0.199**	**0.057**	− **0.344**	− **0.313**	− **0.084**	**0.001**
Triglycerides (mmol/l)	− 1.338	0.911	− 0.168	− 3.148	0.472	0.145
Total cholesterol (mmol/l)	0.727	0.933	0.149	− 1.126	2.581	0.438
LDL (mmol/l)	− 0.491	1.110	− 0.081	− 2.697	1.715	0.659
BP Systolic (mmHg)	− 0.004	0.021	− 0.022	− 0.046	0.037	0.830
HbA_1c_ (%)	− 0.093	0.320	− 0.032	− 0.730	0.544	0.772
IL-6 (pg/ml)	**0.198**	**0.095**	**0.205**	**0.009**	**0.387**	**0.041**
MMP-2 (ng/ml)	− 0.004	0.012	− 0.034	− 0.029	0.020	0.728
MMP-9 (ng/ml)	− 0.002	0.008	− 0.024	− 0.017	0.014	0.819
E-selectin (ng/ml)	0.006	0.036	0.019	− 0.066	0.078	0.865
P-Selectin (ng/ml)	0.026	0.030	0.095	− 0.035	0.086	0.403
VEGF (pg/ml)	0.000	0.027	− 0.001	− 0.053	0.052	0.990

Significant values are in [bold].

**Table 4:** Independent factors for altered CNFD in patients with TIA and stroke.

Dependent variable: CNFD	B	Std. error	Beta	95% CI Lower bound	95% CI Upper bound	Significance
(Constant)	47.556	8.823		30.022	65.091	0.000
Age (years)	− **0.320**	**0.091**	− **0.351**	− **0.500**	− **0.139**	**0.001**
Triglycerides (mmol/l)	− 2.102	1.444	− 0.168	− 4.971	0.767	0.149
Total Cholesterol (mmol/l)	0.902	1.479	0.118	− 2.037	3.840	0.543
LDL (mmol/l)	− 0.975	1.760	− 0.102	− 4.472	2.522	0.581
BP Systolic (mmHg)	− 0.001	0.033	− 0.002	− 0.066	0.064	0.982
HbA_1c_ (%)	− 0.218	0.508	− 0.047	− 1.227	0.791	0.669
IL-6 (pg/ml)	**0.310**	**0.151**	**0.204**	**0.010**	**0.609**	**0.043**
MMP-2 (ng/ml)	− 0.008	0.020	− 0.042	− 0.047	0.031	0.672
MMP-9 (ng/ml)	− 0.003	0.013	− 0.026	− 0.028	0.022	0.803
E-selectin (ng/ml)	0.016	0.057	0.031	− 0.098	0.130	0.779
P-Selectin (ng/ml)	0.003	0.048	0.007	− 0.093	0.099	0.950
VEGF (pg/ml)	0.017	0.042	0.043	− 0.066	0.101	0.682

Significant values are in [bold].

## Discussion

In the present study, we show that patients admitted with TIA or acute ischemic stroke have corneal nerve loss. These data support our previous smaller studies showing a loss of corneal nerves in patients with TIA^23^ and acute ischemic stroke^24^. Moreover, we now show that the severity of corneal nerve loss is comparable between patients with TIA and major stroke. Recently, we have shown greater corneal nerve loss in patients with recurrent stroke, despite comparable vascular risk factors suggesting that assessment of corneal nerve integrity may be a more robust measure of risk of stroke and recurrent stroke compared to the evaluation of conventional risk factors for stroke^28^. Recent skin biopsy studies have also shown a loss of intraepidermal nerve fibres in patients with stroke, particularly those with central post stroke pain^31,32^. Given that we have previously shown comparable reductions in intraepidermal nerves and corneal nerves in diabetic neuropathy^33^, our observations suggest that our finding of corneal nerve loss in TIA and stroke represents more global evidence of nerve loss in stroke and TIA.

Vascular factors play an integral role in the development and progression of ischemic stroke. Mounting evidence shows that circulating biomarkers are associated with incident stroke^7^ and poorer outcomes after stroke^5,6,12^. Endothelial extracellular vesicles have prothrombotic properties and we have recently shown an increase in patients with TIA and ischemic stroke^34^. The current study did not find an elevation in E-selectin, P-selectin or VEGF or a relationship with corneal nerve loss in patients with TIA or stroke. Although, previously, we have shown that corneal nerve fibre loss is independently associated with the presence of white matter hyperintensities in patients with ischemic stroke^26^. We have also shown a reduction in corneal nerves and endothelial cell density in patients with TIA and minor ischemic stroke^23^ and an independent association between corneal endothelial cell density, area and perimeter and acute ischemic stroke^25^.

In the present study, we show that patients with AIS have greater IL-6 and MMP-2 levels compared to TIA which may reflect the severity of neurodegeneration in AIS. The level of IL-6 at admission has been associated with the severity of infarct and neurological outcomes on day 28^35^. Furthermore, the level of IL-6, fibrinogen and white blood cells had a comparable predictive ability to admission NIHSS and infarct size on recovery from stroke at 6-months^36^. It is important to note that the time course for appearance of various biomarkers may vary. Thus, monocyte chemotactic protein-1 (MCP-1), matrix metalloproteinase-9 (MMP-9) and interleukin-6 (IL-6) appear within hours of a stroke, but tissue inhibitor of matrix metalloproteinase-1 (TIMP-1), C-reactive protein (CRP), and S100B appear after 12–24 h and each biomarker had differing abilities to predict 90-day outcomes of stroke^37^. More recently, IL-6 levels correlated with the severity of stroke at admission based on the NIHSS and mRS and also predicted recurrence of stroke^38^. The Reasons for Geographic and Racial Differences in Stroke (REGARDS) study enrolled 30,237 participants and showed that IL-6, but not IL-8 or IL-10 was strongly associated with risk of incident stroke over 5.4 years^39^.

IL-6 has been traditionally considered to be a pro-inflammatory cytokine integral to initiating the acute phase response of the immune system^40,41^. However, it is increasingly recognized as a multifunctional cytokine capable of eliciting both pro- and anti-inflammatory effects^42^. Indeed, in the present study, higher levels of IL-6 were independently associated with a higher CNFD and CNFL, despite a loss of corneal nerves in patients with TIA and stroke. In this context, IL-6 has been associated with axonal regeneration following inflammation^43^ and intrathecal delivery of IL-6 leads to activation of pyramidal cells in the sensory motor cortex following spinal cord injury^44^. In the context of peripheral nerves, IL-6 receptors are expressed on Schwann cells and following nerve transection the administration of IL-6/IL-6R fusion protein resulted in a four-fold increase in myelinated nerve fiber regrowth^45^. IL-6 is also intimately involved in axonal regeneration as it induces increased expression of growth associated protein-43 (GAP-43)^46^. IL-6 deficient mice display reduced amplitude of sensory action potentials and temperature sensitivity and impaired axonal regeneration following sciatic nerve crush injury^47^.

This study has limitations. CCM was not performed in patients with severe disability due to their inability to cooperate during the CCM procedure. This may have biased the outcomes as the results may have been even more pronounced in those with more severe stroke. Second, circulatory markers and neurological outcomes were only measured at one time point.

In conclusion, we show greater corneal nerve loss in patients with stroke and TIA compared to healthy controls and contrary to our initial hypothesis we show that elevated IL-6 levels were independently associated with greater corneal nerve measures. However, to establish a mechanistic link between circulating markers of inflammation and vascular integrity in relation to corneal nerve integrity and outcomes in TIA and stroke, longitudinal studies are required.

## Materials and methods

One hundred and five patients-admitted with AIS (n = 24) and acute ischemic stroke (n = 81) were recruited from the stroke ward at Hamad General Hospital. The diagnosis of TIA and stroke were confirmed clinically and radiologically using AHA criteria^48^. Exclusion criteria included patients with a known history of ocular trauma or surgery, glaucoma, dry eye and corneal dystrophy^49^. Demographic (age, gender, ethnicity) and clinical (blood pressure, HbA_1c_, lipid profile) data were obtained from the admission patients’ electronic health records. All patients underwent assessment of the National Institutes of Health Stroke Scale (NIHSS) at presentation. This study adhered to the tenets of the declaration of Helsinki and was approved by the Institutional Review Board of Weill Cornell Medicine (15-00021) and Hamad General Hospital (15304/15). Informed, written consent was obtained from all patients/guardians before participation in the study.

### Corneal confocal microscopy

All patients underwent CCM (Heidelberg Retinal Tomograph III Rostock Cornea Module; Heidelberg Engineering GmbH, Heidelberg, Germany). CCM uses a 670 nm wavelength helium neon diode laser, which is a class I laser and therefore does not pose any ocular safety hazard. A × 63 objective lens with a numeric aperture of 0.9 and a working distance, relative to the applanating cap (TomoCap; Heidelberg Engineering GmbH) of 0.0 to 3.0 mm, is used. The size of each 2-dimensional image produced is 384 × 384 pixels with a 15° × 15° field of view and 10 μm/pixel transverse optical resolutions. To perform the CCM examination, local anesthetic (0.4% benoxinate hydrochloride; Chauvin Pharmaceuticals, Chefaro, United Kingdom) was used to anesthetize both eyes, and Viscotears (Carbomer 980, 0.2%, Novartis, United Kingdom) was used as the coupling agent between the cornea and the cap. Patients were asked to fixate on an outer fixation light throughout the CCM scan and a CCD camera was used to correctly position the cap onto the cornea^50^. The examination took approximately 10 min for both eyes. The examiners captured images of the central sub-basal nerve plexus using the section mode. Six images per participant were selected based on our established protocol taking into account the position, depth, contrast and focus^51^. All CCM images were manually analyzed using validated, purpose-written software. Corneal nerve fiber density (CNFD), corneal nerve branch density (CNBD) and corneal nerve fiber length (CNFL) were analyzed using CCMetrics (M. A. Dabbah, ISBE, University of Manchester, Manchester, United Kingdom)^16^.

### Blood collection

Following consent, within 48 h of admission, peripheral venous blood (5 mL) from patients was collected in ethylenediaminetetraacetic acid (EDTA) tubes (Vacutainers; Becton Dickinson) by using a 21-gauge needle to reduce platelet stimulation. The tubes containing the blood were immediately transferred on ice to the stroke research laboratory and centrifuged within 60 min at 1500*g* for 15 min at 4 °C to obtain platelet poor plasma. The Plasma was immediately snap frozen and stored at − 80 °C until biomarker quantification. MMP-9, MMP-2, IL-6, E-Selectin, P-Selectin and VEGF-A levels in the plasma were measured by enzyme-linked immunosorbent assay (ELISA) using MMP-9 Human ELISA Kit (Cat: DMP900), MMP-2 Human ELISA Kit (Cat: MMP200), IL-6 Human ELISA Kit (Cat: D6050), E-Selectin Human ELISA Kit (Cat: DSLE00), P-Selectin Human ELISA Kit (Cat: DPSE00) and VEGF Human ELISA Kit (Cat: DVE00). All the ELISA kits were procured from R&D Systems, Inc. Minneapolis MN, USA. The ELISA was performed according to the manufacturer’s instructions.

### Statistical analysis

Statistical justification for the number of participants was based on a power analysis using the freeware program G*Power version 3.0.10 for α (type 1 error) of 0.05 and power (1 − type 2 error) of 0.80 using corneal nerve fibre density mean (37.12 vs 29.18) and standard deviation (8.35 and 7.16) in healthy controls and patients with stroke from our previous study^24^ which established that we needed a minimum of 64 patients with stroke. All statistical analyses were performed using IBM SPSS Statistics software Version 25. Normality of the data was assessed using the Shapiro–Wilk test and by visual inspection of the histogram and a normal Q–Q plot. Data are expressed as mean ± standard deviation (SD). Mann Whitney test (for non-normally distributed variables) and t-test (for normally distributed variables) were performed to find the differences between two groups, and one-way ANOVA was performed to find the differences between three groups and Bonferroni correction was performed. Pearson correlation was performed to find association between corneal nerves and circulatory biomarkers. Multiple linear regression analysis was conducted to evaluate the independent association between CNFL and CNFD and covariates.

### Ethics approval

This study adhered to the tenets of the declaration of Helsinki and was approved by the Institutional Review Board of Weill Cornell Medicine (15-00021) and Hamad General Hospital (15304/15).

### Consent to participate

Informed, written consent was obtained from all patients/guardians before participation in the study.

### Consent for publication

Written consent was obtained from all patients/guardians for publications.

## Data Availability

The datasets generated during and/or analyzed during the current study are available from the corresponding author on reasonable request.
